# Solar Disinfection Improves Drinking Water Quality to Prevent Diarrhea in Under-Five Children in Sikkim, India

**DOI:** 10.4103/0974-777X.68532

**Published:** 2010

**Authors:** BB Rai, Ranabir Pal, Sumit Kar, Dechen C Tsering

**Affiliations:** *Voluntary Health Association of Sikkim (VHAS), Sikkim, India*; 1 *Department of Community Medicine, Sikkim Manipal Institute of Medical Sciences (SMIMS) and Central Referral Hospital (CRH), 5^th^ Mile, Tadong, Gangtok, Sikkim, 737 102, India*; 2 *Department of Microbiology, Sikkim Manipal Institute of Medical Sciences, 5^th^ Mile, Tadong, Gangtok, Sikkim, 737 102, India*

**Keywords:** Diarrhea, SODIS, Solar disinfection, Under-five children

## Abstract

**Background:**

Solar radiations improve the microbiological quality of water and offer a method for disinfection of drinking water that requires few resources and no expertise and may reduce the prevalence of diarrhea among under-five children.

**Aims and Objectives:**

To find out the reduction in the prevalence of diarrhea in the under-five children after consumption of potable water treated with solar disinfection method.

**Materials and Methods:**

This was a population-based interventional prospective study in the urban slum area of Mazegoan, Jorethang, south Sikkim, during the period 1^st^ May 2007 to 30^th^ November 2007 on 136 children in the under-five age group in 102 households selected by random sampling. Main outcome measure was the assessment of the reduction of the prevalence of diarrhea among under-five children after consumption of potable water treated with solar disinfection method practiced by the caregivers in the intervention group keeping water in polyethylene terephthalate (PET) bottles as directed by the investigators. The data were collected by the interview method using a pre-tested questionnaire prepared on the basis of socio-demographics and prevalence of diarrhea. The data were subjected to percentages and chi-square tests, which were used to find the significance.

**Results:**

After four weeks of intervention among the study group, the diarrhea prevalence was 7.69% among solar disinfection (SODIS) users, while 31.82% prevalence was observed among non-users in that period; the reduction in prevalence of diarrhea was 75.83%. After eight weeks of intervention, the prevalence of diarrhea was 7.58% among SODIS users and 31.43% among non-users; the reduction in diarrhea was 75.88% in the study group. The findings were found to be statistically significant.

**Conclusions:**

In our study, we observed that the prevalence of diarrhea decreased significantly after solar disinfection of water was practiced by the caregivers keeping potable water in PET bottles in the intervention group.

## INTRODUCTION

Non-access to safe and potable drinking water is an existent modifiable health risk factor in developing countries like India. Lack of potable water and sanitation causes serious health hazards, predisposing the most vulnerable group, ‘the under-five children,’ to waterborne diseases, which remain a leading cause of illness and death in developing countries. A global estimate suggests that 19% of the total number of child deaths are attributable to diarrhea, and 73% of these deaths are concentrated in 15 developing countries.[1] It is the second commonest cause of death due to infectious diseases in under-five children and also a major cause of adult death in such countries.[2] The occurrence of diarrheal diseases also reflects the economic status of the country and has a complex effect on the country’s economy by adversely affecting the health of its work force.[3]

Solar disinfection (SODIS) is a safe method to improve the quality of drinking water by simply using solar radiation to inactivate agents causing diarrheal diseases. The most favorable region for SODIS lies between latitude 15°N to 35°S. The semi-arid regions are characterized by high solar radiation with limited clouds and rainfall. SODIS utilizes UV-A radiations (wavelengths, 320-400 nm), which react with dissolved oxygen in the water. This results in formation of free radicals of oxygen and hydrogen peroxide, which sterilize water. SODIS is a method of disinfecting water using only sunlight and plastic PET bottles. SODIS is a cheap and effective method for decentralized water treatment, usually applied at the household level, and is recommended by the World Health Organization as a viable method for household water treatment and safe storage. It has been shown that the SODIS method (and other methods of household water treatment) can very effectively remove pathogenic contamination of water. However, infectious diseases are also transmitted through other pathways as a result of general lack of sanitation and hygiene. Studies on the reduction of diarrhea among SODIS users show reduction values of 30% to 80%.[4–7] The solar disinfection unit has been field-tested by Centro Panamericano de Ingenieria Sanitariay Ciencias del Ambiente in Lima, Peru. At moderate light intensity, the solar disinfection unit was capable of reducing the bacterial load in a controlled contaminated water sample by 4 log10 U and disinfected approximately 1 L of water in 30 minutes.[8]

Pooling data from meta-analysis suggests that interventions to improve the microbiological quality of drinking water are effective in preventing diarrhea, both for populations of higher ages and children less than five years old. Subgroup analyses suggest that household interventions are more effective in preventing diarrhea than interventions at the water source. Effectiveness was positively associated with compliance.[9]

The study was undertaken to find out the reduction in the prevalence of diarrhea in the under-five children after consumption of potable water treated with solar disinfection method. So far there has been no study done in this field in the state; and to the best of our knowledge, this was one of the first few studies reported from the northeastern part of India.

## MATERIALS AND METHODS

### Settings and design

A population-based interventional prospective study in the urban slum area of Mazegoan, Jorethang, south Sikkim, was conducted during the period 1^st^ May 2007 to 30^th^ November 2007.

### Participants

House-to-house survey was conducted in urban areas of Mazegoan under Jorethang, south Sikkim. Of the total 416 households, 102 households were identified having one or more under-five children, accounting for a total of 136 under-five children. These households were grouped as study and control groups. Fifty-two households with 65 under-five children were selected randomly from among the target population and subjected to study intervention.

### Interventions

Solar disinfection of water was practiced by the caregivers keeping water in PET bottles in the intervention group as directed by the investigators.

### Study instrument

The data collection tool used for the study was an interview schedule that was developed at the healthcare facility with the assistance of the faculty members and other experts from the health department of the Government of Sikkim. The close-ended questionnaire contained questions relating to prevalence of diarrhea in Sikkim. By initial translation, back-translation, re-translation followed by pilot study, the questionnaire was custom-made for the study. The pilot study was carried out at the Jorethang primary health center (PHC) among general patients, following which some of the questions from the interview schedule were modified.

### Main outcome measures

Reduction in the prevalence of diarrhea in under-five children

### Sampling frame and data collection procedure

The state of Sikkim has four districts, namely, East (Gangtok), West (Gyalshing), South (Namchi) and North (Mangan). South District was selected by simple random sampling (lottery method). The ethical permission to conduct the study in the urban slum area of Mazegoan at Jorethang in south Sikkim was taken from the Office of the Chief Medical Officer, south Sikkim, Health Care, Human Services and Family Welfare Department, Government of Sikkim. The health workers of the field partner, PUMASS, informed and motivated the intervention group along with the intervention. All the participants were explained about the purpose of the study and were ensured strict confidentiality, and then verbal informed consent was taken from each of them before the starting of the procedure. Information on SODIS was disseminated during health education sessions to complement the findings of the study. We collected samples of water from where the caregivers were collecting drinking water, viz., Rangit River and spring water. Bacteriological testing was undertaken at the STNM Hospital, Gangtok, approximately 50 km away from the study area. Water samples were collected from sources of water and from the SODIS users in sterile plastic bottles and were tested for coliform organism by the ‘most probable number’ technique.[9] According to the guidelines of the Bureau of Indian Standards, samples with 0 coliform/100 mL of water were considered excellent; with 1-10 coliform(s), as satisfactory; and above 10 coliforms, as unsatisfactory.[10] We had taken utmost care in double-blinding at all stages. Blinding (double) was done through coding the samples of water; and also during collection of data regarding prevalence of diarrhea, separate groups of volunteers were engaged. Bias was taken care of by analysis of reduction of the prevalence of diarrhea only in under-five children, as they have the lowest probability to get enteric infection by consuming water outside their home.

### Statistical analysis

Percentages and Chi-square tests were used in this study to calculate the significance of reduction in the prevalence of diarrhea in the under-five children after consumption of potable water treated with solar disinfection method.

## RESULTS

In the 102 households identified having one or more under-five children, there were 136 under-five children, who were grouped as study and control groups. After four weeks of intervention among the study group, the diarrhea prevalence was 7.69% among SODIS users and 31.82% among non-users in the last one month (x^2^ = 10.69; d.f.= 1; *P*<0.05). The reduction in diarrhea was 75.83%. After eight weeks of intervention, the prevalence of diarrhea was 7.58% among SODIS users and 31.43% among non-users (x^2^ = 11.25; d.f.= 1; *P*<0.05). The reduction in diarrhea was 75.88% in the study group.

## DISCUSSION

In our study, we observed thatdiarrhea prevalence was decreased significantly among users of water disinfected using the solar disinfection (SODIS) method practiced by the caregivers keeping water in PET bottles in the intervention group.

Study at Kenya shows that among the 108 children in households allocated for solar water treatment, diarrhea was reported in 439 two-week reporting periods during the 12-week trial [average, 4.1 (SD, 1.2) per child]. By comparison, the 98 children in the control households reported diarrhea during 444 two-week reporting periods [average, 4.5 (SD, 1.2) per child]. Diarrhea severe enough to prevent performance of duties occurred during 186 reporting periods in the solar group and during 222 periods in the control group [average, 1.7 (SD, 1.2) *vs*. 2.3 (SD, 1.4)]. After adjustment for age, solar treatment of drinking water was associated with a reduction in all diarrhea episodes [odds ratio, 0.66 (0.50-0.87)] and in episodes of severe diarrhea [0.65 (0.50-0.86)].[4]

Researchers from Kajiado district, Kenya, reported that children who were drinking solar-disinfected water had a significantly lower risk of severe diarrheal disease over 8,705 two-weekly follow-up visits; two-week prevalence was 48.8% compared with 58.1% in controls, corresponding to an attributable fraction of 16.0%. While this reduction is modest, it was sustained over a year.[5]

Study from Kenya proved that among 131 households in the trial area, of which 67 had been randomized to solar disinfection, there was no significant difference in the risk of cholera in adults or in older children in households randomized to solar disinfection. However, there were only three cases of cholera in the 155 children aged less than six years drinking solar-disinfected water compared with 20 of 144 controls.[6]

In an intervention study conducted in an urban slum of Vellore town, there was significant reduction in the incidence, duration and severity of diarrhea in children receiving solar-disinfected water, despite 86% of the children drinking water other than that treated by the intervention. The incidence of diarrhea in the intervention group was 1.7 per child-year; and among controls, 2.7 per child-year, with an incidence rate ratio of 0.64 (95% CI, 20.48-0.86). The risk of diarrhea was reduced by 40% by using solar disinfection. In qualitative evaluation of acceptability, most women felt that solar disinfection was a feasible and sustainable method of disinfecting water.[7]

A cluster-randomized controlled trial in 22 rural communities in Bolivia evaluated the effect of SODIS in reducing diarrhea among children under the age of five years. A local nongovernmental organization conducted a standardized interactive SODIS-promotion campaign in 11 communities, targeting households, communities and primary schools. Within the intervention arm, 225 households (376 children) were trained to expose water-filled PET bottles to sunlight. Eleven communities (200 households, 349 children) served as a control. Mean compliance with SODIS was 32.1%. The reported incidence rate of gastrointestinal illness in children in the intervention arm was 3.6 as compared to 4.3 episodes among the control group. The relative rate of diarrhea adjusted for intracluster correlation was 0.81 (95% confidence interval, 0.59-1.12). The median length of diarrhea was three days in both groups.[10]

In spite of our best efforts, the number of participants in the study sample was small as we had to face tough opposition while convincing the apparently healthy folk in the urban slum regarding the effectiveness of a simple cost-effective method. Moreover, sparsely spread population in hilly terrains was another hindrance, and constant motivation was needed so as to prevent attrition during the study.

### The strength of the study

The strength of the study was that it studied a novel cost-effective technology using the solar radiations to improve the microbiological quality of water, and offer a method for disinfection of drinking water that requires few resources and no expertise and may reduce the prevalence of diarrhea among under-five children. Bias was taken care of by analysis of reduction of the prevalence of diarrhea only in under-five children, as they have the lowest probability to get enteric infection by consuming water outside their home. So far there has been no study done in this field in the state; and to the best of our knowledge, this was one of the first few studies reported from the northeastern part of India. Through repeated motivation, we could have managed complete cooperation and full participation from the study population. Hence the compliance rate need not have been calculated

### The limitations of the study

SODIS does not change the chemical quality of water and requires relatively clear water (turbidity, less than 30 NTU) for effective penetration of UVA from sunlight. SODIS requires suitable weather conditions; continuous rainfall and clouds affect the disinfection process. Moreover, SODIS is not useful to treat large volumes of water; in addition, transparent PET container is needed. We could not study seasonal variations.

### Future directions on the basis of this study

Solar radiations reduce the microbial load of water and offer a method for disinfection of drinking water that requires few resources and no expertise and may reduce the prevalence of diarrhea among under-five children by inactivating the agents causing diarrheal diseases. We hope to find out the extent to which this cost-effective novel technology will be implemented in future in this remote northeastern hill state of Sikkim. The key factors remain ‘motivation’ and ‘belief.’ We have to generate awareness among our peers, public health experts, health services researchers, healthcare providers and planners so that they accept and implement solar disinfection as a simple and cost-effective technology to improve quality of drinking water and to reduce the incidence of diarrhea in under-five children, which is still a public health problem in developing countries like India.

## CONCLUSION

The study was undertaken to find out whether this cost-effective, novel technology could have been implemented in this remote northeastern hill state of Sikkim. In our study, we observed that the prevalence of diarrhea decreased significantly after solar disinfection of water was practiced by the caregivers keeping potable water in PET bottles in the intervention group. These findings suggest that solar disinfection of water may reduce diarrhea in communities with no access to other means of disinfection. The ‘social vaccine’ of health empowerment, along with acceptance of appropriate technology as a component of overall development by the rural and urban health personnel (Department of Health and Family Welfare); with an additional input of health awareness and motivation by Anganwadi workers (Department of Women and Child Development), elected women representatives in the *panchayats*(Department of Rural Development and Panchayati Raj) and nongovernmental development agencies could be a collaborative effort towards decreasing the prevalence of diarrhea. This could lay the foundation for the introduction of primary health care with community participation.

There is need for changes in policies, standards, quality control, monitoring and evaluation and integration of newer alternatives in public health: a) to address unprivileged urban slum folk; b) ensuring extensive motivation as well as a comparable quality of coverage in every state; c) to use behavior-change communication strategies matching healthcare resources. In countries like India, with a great regional heterogeneity, this technology holds the potential to be used as a tool to prevent diarrheal deaths in the under-five children.

**Table 1:** Prevalence of diarrhea among underfive children who used SODIS and those who did not (after four weeks of intervention)

	No. of households	No. of children	No. of diarrheal cases
SODIS users	52	65	05 (7.69)
SODIS non-users	50	66	21 (31.82)

Figures in parenthesis are in percentage

**Table 2:** Prevalence of diarrhea among underfive children who used SODIS and those who did not (after eight weeks of intervention)

	No. of households	No. of children	No. of diarrheal cases
SODIS users	52	66	05 (7.58)
SODIS non-users	50	70	22 (31.43)

Figures in parenthesis are in percentage
